# Senolytic Therapy for Cerebral Ischemia-Reperfusion Injury

**DOI:** 10.3390/ijms222111967

**Published:** 2021-11-04

**Authors:** Songhyun Lim, Tae Jung Kim, Young-Ju Kim, Cheesue Kim, Sang-Bae Ko, Byung-Soo Kim

**Affiliations:** 1 School of Chemical and Biological Engineering, Seoul National University, Seoul 08826, Korea; petitrsh@snu.ac.kr (S.L.); cheesue@snu.ac.kr (C.K.); 2 Department of Neurology, Seoul National University Hospital, Seoul 03080, Korea; ttae35@gmail.com (T.J.K.); biokyj@hanmail.net (Y.-J.K.); 3 Department of Critical Care Medicine, Seoul National University Hospital, Seoul 03080, Korea; 4 Interdisciplinary Program for Bioengineering, Seoul National University, Seoul 08826, Korea; 5 Institute of Chemical Processes, Seoul National University, Seoul 08826, Korea; 6 Institute of Engineering Research, Seoul National University, Seoul 08826, Korea; 7 Bio-MAX Institute, Seoul National University, Seoul 08826, Korea

**Keywords:** ABT263, astrocyte, inflammation, ischemia-reperfusion injury, ischemic stroke, senescence, senolytic therapy

## Abstract

Ischemic stroke is one of the leading causes of death, and even timely treatment can result in severe disabilities. Reperfusion of the ischemic stroke region and restoration of the blood supply often lead to a series of cellular and biochemical consequences, including generation of reactive oxygen species (ROS), expression of inflammatory cytokines, inflammation, and cerebral cell damage, which is collectively called cerebral ischemia-reperfusion (IR) injury. Since ROS and inflammatory cytokines are involved in cerebral IR injury, injury could involve cellular senescence. Thus, we investigated whether senolytic therapy that eliminates senescent cells could be an effective treatment for cerebral IR injury. To determine whether IR induces neural cell senescence in vitro, astrocytes were subjected to oxygen-glucose deprivation/reoxygenation (OGD/R). OGD/R induced astrocyte senescence and senescent cells in OGD/R-injured astrocytes were effectively eliminated in vitro by ABT263, a senolytic agent. IR in rats with intraluminal middle cerebral artery occlusion induced cellular senescence in the ischemic region. The senescent cells in IR-injured rats were effectively eliminated by intravenous injections of ABT263. Importantly, ABT263 treatment significantly reduced the infarct volume and improved neurological function in behavioral tests. This study demonstrated, for the first time, that senolytic therapy has therapeutic potential for cerebral IR injury.

## 1. Introduction

Stroke is a leading cause of mortality and morbidity and is associated with a high disease burden because of disabilities related to the sequelae of stroke. Approximately 80% of strokes are ischemic strokes that are caused by the occlusion of a cerebral artery [1,2,3]. Ischemic stroke leads to cellular dysfunction and cell death in the ischemic region of the brain [4,5]. Restoration of blood supply by reperfusion can salvage ischemic tissue but reperfusion per se causes tissue injury, which is called cerebral ischemia-reperfusion (IR) injury [4,5]. As endovascular therapy for ischemic stroke has advanced in recent years, the challenge of devising neuroprotective strategies for cerebral IR injury has grown. The pathology of cerebral IR injury involves the generation of excessive reactive oxygen species (ROS), oxidative damage to cells, activation of the inflammatory cascade reaction, and induction of cellular apoptosis in reperfused tissue [6,7,8,9]. Several studies have demonstrated the detrimental effects of ROS-induced damage on long-term stroke outcomes and redox imbalance-mediated impairment of neurobehavioral functions in cerebral IR-injured animals [10,11]. Thus, ROS inhibition and maintenance of redox balance would carry significant therapeutic benefit in cerebral IR injury.

Cellular senescence is a process in response to various cellular stresses and is characterized by irreversible cell cycle arrest, apoptosis resistance, and the expression of proinflammatory cytokines known as the senescence-associated secretory phenotype (SASP) [12,13,14]. Senescence contributes to various age-related or degenerative diseases by senescent cell accumulation and SASP-mediated inflammation and tissue damage [13,15,16]. In addition to age-related or degenerative diseases, it has also been shown that cardiac IR injury induces senescence of cardiac cells [17]. An increase in ROS levels is one of the main mechanisms by which cellular senescence is induced. ROS activate p16 and p53 through the kinase effector p38, which leads to cellular senescence through downstream mediators [15]. Senolytic drugs selectively kill senescent cells by targeting abnormally increased protein expression in the anti-apoptosis pathway activated by senescence [18]. Senolytic therapy that targets and eliminates senescent cells using senolytic drugs has been reported as an effective treatment for various degenerative diseases, including Alzheimer’s disease, in animal models [17,19,20,21,22]. Here we hypothesized that cellular senescence could be a key driver involved in the pathogenesis of cerebral IR injury. Therefore, elimination of senescent cells in brain tissue induced by ROS following cerebral IR could be a therapeutic target for cerebral IR injury.

In this study, ABT263 (navitoclax, anti-cancer drug), which selectively induces apoptosis of senescent cells by disrupting interactions within Bcl-2/Bcl-xL and pro-death proteins, was used as a senolytic drug [23]. We investigated whether cerebral IR injury induces neural cell senescence in vitro and in vivo. We also evaluated the therapeutic effect of ABT263 in senescent cells and IR-injured rat brains. By ABT263 treatment, senescent cells were eliminated in vitro and in ischemic region of brain tissue. Furthermore, injections of ABT263 significantly reduced infarct tissue volume and improved neurological outcome.

## 2. Results

### 2.1. In Vitro Induction of Senescence in Rat Cortex Astrocytes by Oxygen-Glucose Deprivation/Reoxygenation (OGD/R)

To evaluate the cytotoxicity of OGD/R on rat cortex astrocytes, cell viability and cell death were determined after OGD/R treatment (Figure S1). Cell viability evaluated by 3-(4,5-dimethylthiazol-2-yl)-2,5-diphenyltetrazolium bromide (MTT) assay was not significantly different between normal astrocytes (NA) and astrocytes subjected to OGD/R (OGD/R-A) at both low (5 × 10^3^ cells cm^−2^) and high (1 × 10^4^ cells cm^−2^) cell densities, regardless of the concentration of serum. However, the results of the lactate dehydrogenase (LDH) release assay showed that at both cell densities, OGD/R treatment with medium containing 1% serum showed a cytotoxic effect on astrocytes, while OGD/R treatment with 10% serum-containing medium showed no significant effect (Figure S1). Senescence-associated beta-galactosidase (SA-β-gal) staining was performed to determine whether senescence was induced by OGD/R at both low and high densities of cells in 1% and 10% serum-containing medium (Figure S1). Senescence was induced in astrocytes subjected to OGD/R in 10% serum-containing medium irrespective of cell density. To effectively induce senescence without inducing extensive cell death, cells seeded at a low density (5 × 10^3^ cells cm^−2^) were used and OGD/R treatment with 10% serum-contained medium was performed in subsequent experiments.

**Figure 1:**
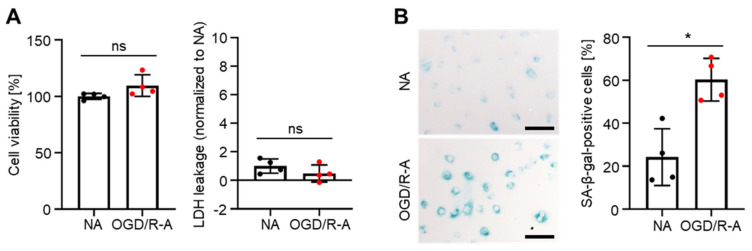
Rat astrocyte senescence induced in vitro by oxygen-glucose deprivation/reoxygenation (OGD/R). (**A**) Viability and death of normal astrocytes (NA) and OGD/R-injured astrocytes (OGD/R-A), as evaluated by 3-(4,5-dimethylthiazol-2-yl)-2,5-diphenyltetrazolium bromide (MTT) assay and lactate dehydrogenase (LDH) release assay, respectively. OGD/R injury showed no cytotoxicity to rat cortex astrocytes. ns = not significant. *n* = 4 per group. (**B**) Representative images and quantification of senescence-associated beta-galactosidase (SA-β-gal) staining (green) after 4 h of OGD/R injury. OGD/R injury induced senescence in rat cortex astrocytes. Scale bars, 100 µm. *n* = 4 per group. (**A**,**B**) Data are presented as mean ± SD. * *p* < 0.05. Mann–Whitney test was used for statistical analysis.

Both cell viability and LDH leakage were not significantly different between normal astrocytes (NA) and astrocytes subjected to OGD/R (OGD/R-A) in Figure 1A. The data indicated that in vitro OGD/R treatment was not cytotoxic to rat cortex astrocytes. SA-β-gal staining showed that senescence was induced by OGD/R (Figure 1B). OGD/R increased the percentage of senescent cells (60%) compared to normal astrocytes (22%). An average of 22% of normal cells were senescent, which might be due to senescence induction by the culture of primary cells [24]. The data indicate that OGD/R induces senescence in rat cortex astrocytes.

### 2.2. Selective Elimination of Senescent Astrocytes by ABT263 In Vitro

**Figure 2:**
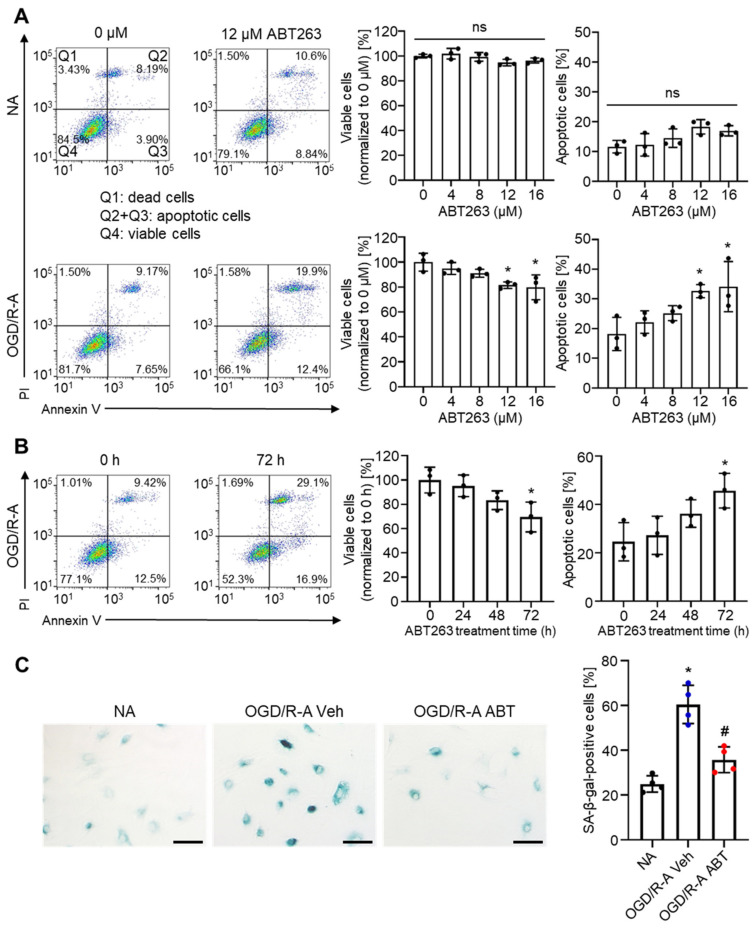
Selective removal of senescent astrocytes by ABT263 in vitro. (**A**) Flow cytometric plots of normal astrocytes (NA) and OGD/R-injured astrocytes (OGD/R-A), showing selective induction of OGD/R-A apoptosis by ABT263. (**B**) Flow cytometric plots of OGD/R-A treated with 12 µM ABT263 for different periods of time. To effectively remove senescent astrocytes, the duration period of ABT263 treatment should be at least 72 h. (**C**) SA-β-gal staining of NA and OGD/R-A treated with either vehicle or 12 µM ABT263 for 96 h, showing removal of senescent cells of OGD/R-A. Veh indicates vehicle treatment, and ABT indicates ABT263 treatment. Scale bars, 100 µm. *n* = 4 per group. * *p* < 0.05 compared to NA, # *p* < 0.05 compared to OGD/R-A Veh. (**A**,**B**) *n* = 3 per group. * *p* < 0.05 compared to 0 µM or 0 h. (**A**–**C**) Data are presented as mean ± SD. One-way ANOVA with Tukey’s post-test was used for statistical analysis.

Next, we investigated whether ABT263 can selectively eliminate senescent astrocytes. Astrocytes seeded at a low density (5 × 10^3^ cells cm^−2^) and subjected to OGD/R treatment with 10% serum-containing medium were used as OGD/R-injured (senescence-induced) astrocytes (OGD/R-A). Previous studies have demonstrated that ABT263 can selectively eliminate senescent cells in several organs [25,26]. To evaluate both the therapeutic effects of ABT263 concentration by the in vitro removal of senescent cells and its possible off-target cytotoxicity on nonsenescent normal cells, normal astrocytes (NA) and OGD/R-A were treated with ABT263 at various concentrations for 24 h (Figure 2A). Then, flow cytometry analysis was performed after annexin V-propidium iodide (PI) staining. The percentage of viable cells (annexin V-PI−) and apoptotic cells (annexin V+PI−) did not change in the normal astrocytes following treatment with ABT263 at various concentrations. In contrast, in OGD/R-A cells, the ratio of viable cells (annexin V-PI−) decreased and the ratio of apoptotic cells (annexin V+PI−) increased as ABT263 concentration increased. In the OGD/R-A group, ABT263 treatment at concentrations higher than 12 µM exhibited statistically significant effects on cell viability and apoptosis compared to the no treatment group. These data indicated that ABT263 could selectively remove senescent astrocytes by inducing apoptosis in OGD/R-A cells.

To examine the effect of ABT263 treatment duration on the in vitro removal of senescent cells, OGD/R-A were treated with 12 µM ABT263 for various time periods (Figure 2B). The percentage of viable cells (annexin V-PI−) was significantly decreased, and the per-centage of apoptotic cells (annexin V+PI−) was significantly increased by ABT263 treatment for 72 h. These data indicate that the ABT263 treatment duration period should be at least 72 h to effectively remove senescent astrocytes.

To confirm that ABT263 treatment can eliminate senescent astrocytes in vitro, astrocytes were treated with either vehicle or 12 µM ABT263 for 96 h, followed by SA-β-gal staining (Figure 2C). Sixty percent of OGD/R-A following vehicle treatment were SA-β-gal-positive (senescent cells). ABT263 treatment of OGD/R-A significantly reduced the percentage of SA-β-gal-positive cells to 36%, which was not significantly different from that of normal healthy astrocytes.

### 2.3. Elimination of Senescent Cells and Attenuation of Inflammation by ABT263 in Cerebral IR-Injured Middle Cerebral Artery Occlusion (MCAO) Rats

**Figure 3:**
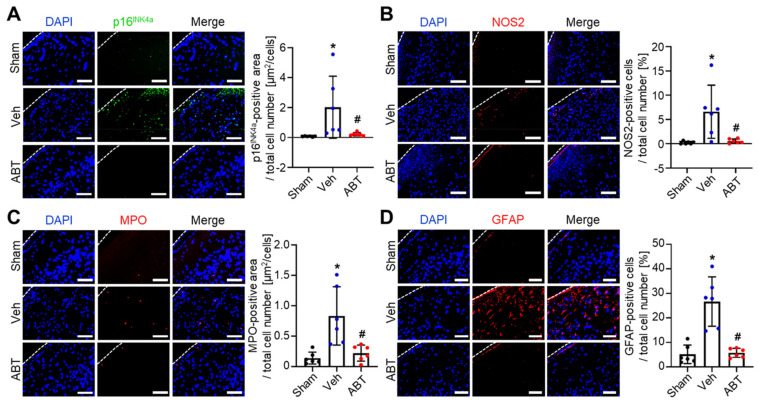
Removal of senescent cells and attenuation of inflammation in ischemic stroke lesion by ABT263 in middle cerebral artery occlusion (MCAO) ischemia-reperfusion rat model. MCAO rats were treated with intravenous injection of either vehicle (Veh) or ABT263 (ABT). The sham group did not receive MCAO. Immunostaining for (**A**) p16^INK4a^, (**B**) NOS2, (**C**) MPO and (**D**) GFAP in the ischemic stroke lesion of MCAO rats on day 4. (**A**–**D**) Scale bars, 100 µm. *n* = 6 per group. Data are presented as mean ± SD. * *p* < 0.05 compared to Sham, # *p* < 0.05 compared to Veh. One-way ANOVA with Tukey’s post-test was used for statistical analysis.

ABT263 was intravenously injected three times: immediately after reperfusion, 1 day after reperfusion, and three days after reperfusion. Ischemic stroke regions were analyzed on Day 4. p16^INK4a^ immunostaining indicated that IR in rats with MCAO induced cerebral senescence (Figure 3A). p16^INK4a^ immunostaining also revealed that senescent cells in the IR-injured MCAO rats could be eliminated by ABT263. The in vivo dose used in this study was determined by considering the route and frequency of administration in previous studies using ABT263 as a senolytic for other diseases [17,20,21,22,27].

Immunostaining of the ischemic stroke regions showed that the expression of nitric oxide synthase 2 (NOS2), a proinflammatory cytokine and SASP, was significantly increased during cerebral IR injury and decreased by ABT263 treatment (ABT) (Figure 3B). The expression of myeloperoxidase (MPO), a marker of neutrophil activation, was also significantly increased during cerebral IR injury and decreased by ABT263 treatment (Figure 3C). Moreover, immunostaining of glial fibrillary acidic protein (GFAP) showed that reactive astrocytes in the ischemic stroke regions were significantly increased during cerebral IR injury but decreased following ABT263 treatment (Figure 3D). Collectively, these data indicate that ABT263 can effectively remove senescent cells and attenuate inflammation in the ischemic stroke region, both of which are induced by cerebral IR injury.

### 2.4. Therapeutic Effects of ABT263 in Cerebral IR-Injured MCAO Rats

**Figure 4:**
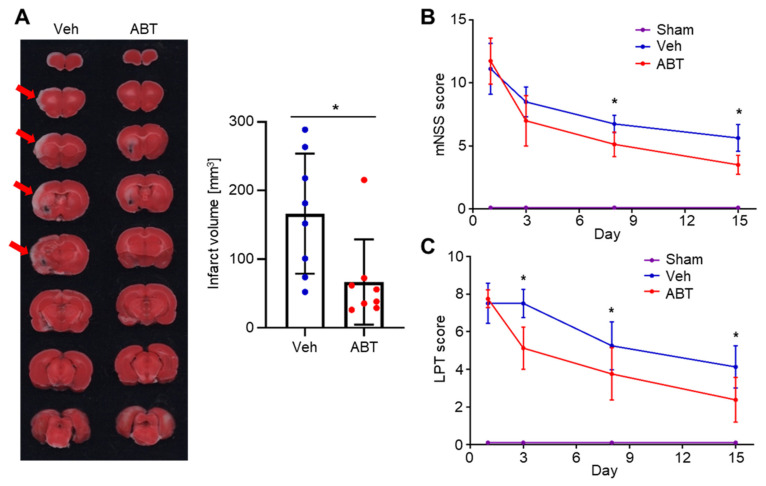
Therapeutic effect of ABT263 in MCAO ischemia-reperfusion rat model. MCAO rats were treated with intravenous injections of either vehicle (Veh) or ABT263 (ABT). The sham group did not receive MCAO. (**A**) 2,3,5-triphenyltetrazolium chloride (TTC) staining of serial sections of the brain and quantification of the cerebral infarct volume on day 4. The ABT263 treatment group had significantly smaller infarct volume compared to the Veh group. Arrows indicate cerebral infarct. *n* = 8 per group. * *p* < 0.05. Data are presented as mean ± SD. Mann–Whitney test was used for statistical analysis. Brain functional recoveries of MCAO rats evaluated by (**B**) the limb placing test (LPT) and (**C**) modified neurological severity score (mNSS). The mNSS tests and the limb-placing tests showed that ABT263 treatment significantly improved neurological functions after MCAO. *n* = 6 per group. Data are presented as mean ± SD. * *p* < 0.05 compared to Veh or Sham. Two-way repeated measures ANOVA was used for statistical analysis.

ABT263 treatment significantly decreased the infarct volume compared to vehicle treatment (166.2 ± 87.4 versus 66.7 ± 62.2 mm^3^, *p* < 0.05) (Figure 4A). We also evaluated whether ABT263 treatment improved neurological functions. The sham group, which did not receive MCAO, scored 0 in both modified neurological severity score (mNSS) tests and limb-placing tests. The mNSS tests revealed that ABT263 treatment significantly improved neurological functions after Day 8 (Figure 4B). Similarly, the limb-placing tests indicated a significant improvement in neurological functions from Day 3 by ABT263 treatment (Figure 4C).

## 3. Discussion

This study demonstrated that ABT263, a senolytic drug, has therapeutic potential for the treatment of cerebral IR injury. Here, we showed that cerebral IR injury induces cellular senescence in the ischemic stroke region. We also showed that ABT263 injection could eliminate senescent cells and attenuate cerebral inflammation following cerebral IR injury, leading to improved neurological functions.

In this study, we used OGD/R to investigate whether cerebral IR injury induces senescence in the brain using astrocytes in vitro as they are the most abundant nonneuronal cells the brain [28,29,30]. They play crucial roles in maintaining normal brain function since they communicate with various types of cells in brain tissue such as neurons and brain endothelial cells [28,30]. For these reasons, astrocytes have been widely used in previous in vitro studies on ischemic stroke or hemorrhagic stroke [28,31,32]. Thus, it would be appropriate to consider astrocytes as a representative pathological target in studies on cerebral IR injury. In this study, we found that rat cortex astrocytes subjected to OGD/R underwent senescence (Figure 1B).

SA-β-gal and p16^INK4a^ were used as senescence markers to identify senescent cells in this study. SA-β-gal staining can be used to detect senescent cells both in vitro and in vivo [33,34]. p16^INK4a^ is a cell cycle inhibitor also known as cyclin-dependent kinase inhibitor 2a [15,35]. It induces early senescence in cells through its sustained activation [14]. Both SA-β-gal and p16^INK4a^ have been widely used to identify senescent cells in several previous studies [17,19,20,21,22,27,36].

The SASP is a complex proinflammatory response initiated by senescent cells [15]. It includes the secretion of various proinflammatory cytokines, such as IL-1α/β, IL-6 and IL-8, and even nonprotein soluble factors, such as nitric oxide and ROS [15,37]. SASP components secreted by senescent cells often cause inflammation and trigger senescence in contiguous cells [15]. The production of nitric oxide, which is a SASP component, is associated with the consequences of ischemic stroke. Cerebral ischemia results in NOS2 activation, leading to the production of nitric oxide which induces necrosis and apoptosis in the ischemic region [5]. Therefore, the production of nitric oxide, which is a SASP component or a product of ischemia, is associated with the consequences of ischemic stroke. Additionally, NOS2 is a functional marker of M1-type microglia/macrophages [38,39,40]. Thus, the decreased levels of NOS2 in the ischemic region (Figure 3B) which resulted from ABT263 injection into cerebral IR-injured MCAO rats, indicates attenuation of cerebral inflammation.

In the initial stages of inflammation, neutrophil chemotactic factors are produced, which recruit and activate neutrophils [41,42,43]. As major effector cells involved in tissue damage within cerebral IR injury, neutrophils express MPO, an enzyme that provokes tissue damage via oxidant production [44]. Therefore, the decrease in MPOs in the ischemic region (Figure 3C), which resulted from ABT263 injection into cerebral IR-injured MCAO rats indicates attenuation of the neutrophil-mediated early proinflammatory response. In the early phases of cerebral IR injury, astrocytes are activated by proinflammatory cytokines which include several SASP components [15,28,45]. Activated astrocytes secrete proinflammatory cytokines, inducing neuroinflammation [28]. Therefore, the reduced expression of GFAP (Figure 3C), a marker of astrocyte activation [45,46,47], which resulted from ABT263 injection into cerebral IR-injured MCAO rats, also indicates the attenuation of neuroinflammation.

Our data regarding the therapeutic potential of ABT263 in the treatment of cerebral IR injury are consistent with the therapeutic efficacy of ABT263 in the treatment of cardiac IR injury in a previous study [17]. This study demonstrated that cardiac IR injury induces senescence in rat hearts and that ABT263 treatment can eliminate senescent cells, ameliorate inflammatory responses and attenuate adverse cardiac remodeling following cardiac IR injury. IR induces a series of oxidative stresses, proinflammatory cytokine secretion, inflammation, and tissue damage [48]. Thus, the previous study on ABT263 treatment for cardiac IR injury gives further credence to the therapeutic potential of ABT263 treatment for cerebral IR injury.

There were some limitations to this study. First, ABT263-treated cells tested in vitro included only astrocytes, which are the most commonly used cell type for in vitro study of ischemic stroke. Other cell types such as neuronal cells, microglia, endothelial cells and pericytes, are also crucial mediators of the pathological changes that occur after cerebral IR injury [49,50,51]. Additional studies are required to investigate the senescence of these cells and their potential roles in cerebral IR injury. Second, we used transient MCAO for ischemic-reperfusion injury, which induced an area of ischemic damage in the MCA territory smaller than that of other studies. Third, clinical studies have revealed that systemic administration of ABT263 often causes systemic side effects such as thrombocytopenia, a rapid drop in platelet count [52,53]. Therefore, additional studies are required to develop a system for the local delivery of senolytic drugs to avoid the potential risk of systemic toxicity.

In conclusion, this study demonstrated that intravenous ABT263 treatment attenuated inflammation through elimination of senescent cells and improved functional outcomes after IR in the brain. ABT263, a senolytic drug, is a novel therapeutic candidate for cerebral ischemia with IR injury.

## 4. Materials and Methods

### 4.1. Cell Culture

Rat brain cortex astrocytes (R-CxAs-520, Lonza, Basel, Switzerland) were cultured in astrocyte growth medium with supplements (CC-3186, Lonza) at 37 °C with 5% CO_2_. Cell passaging was performed when a monolayer of adherent cells reached 80% confluence. Cells at the sixth passage were used for further experiments.

### 4.2. Oxygen-Glucose Deprivation/Reoxygenation (OGD/R)

Rat brain cortex astrocytes were subjected to OGD/R as described previously [28,54]. Briefly, cells were seeded at a density of either 5 × 10^3^ cells cm^−2^ (low cell density) or 1 × 10^4^ cells cm^−2^ (high cell density) and cultured in Dulbecco’s modified Eagle’s medium (DMEM, Sigma-Aldrich, St. Louis, MO, USA) with no glucose supplemented with 100 U/mL penicillin and 100 µg/mL streptomycin (P/S; Gibco, Waltham, MA, USA) and 1% or 10% (*v*/*v*) fetal bovine serum (FBS; Gibco) in an incubator filled with 1% O_2_, 5% CO_2_, and 94% N_2_ at 37 °C. After 4 h, oxygen-glucose deprivation was terminated by changing the medium to DMEM with high glucose (Gibco) supplemented with 10% (*v*/*v*) FBS and P/S under normal conditions (37 °C with 95% air and 5% CO_2_) for 20 h. Cells in the normal group were cultured in DMEM with high glucose supplemented with 10% (*v*/*v*) FBS and P/S under normal conditions.

### 4.3. 3-(4,5-Dimethylthiazol-2-yl)-2,5-Diphenyltetrazolium Bromide (MTT) Assay and Lactate Dehydrogenase (LDH) Assay

To measure cell viability, an MTT assay was performed using EZ-cytox (DoGen, Seoul, South Korea) according to the manufacturer’s instructions. Briefly, EZ-cytox solution with fresh culture medium was added to cell cultures and maintained for 2 h at 37 °C. The optical density at 450 nm was measured using a Powerwave X340 microplate reader (BIO-TEK Instruments, Winooski, VT, USA). To measure the cytotoxicity, LDH release was measured by an LDH assay kit (EZ-LDH, DoGen) according to the manufacturer’s instructions. Briefly, culture medium collected from cell cultures was mixed with the LDH assay solution and maintained for 30 min at room temperature. The optical density at 450 nm was measured, and the percentage of LDH release was calculated.

### 4.4. Senescence-Associated Beta-Galactosidase (SA-β-Gal) Staining

To stain senescent cells, an SA-β-gal staining kit (#9860, Cell Signaling Technology, Danvers, MA, USA) was used according to the manufacturer’s instructions. Briefly, cells were fixed in a fixative solution for 15 min and incubated in β-galactosidase staining solution adjusted to pH 6 in a dry incubator overnight. The cells were imaged under light microscopy (IX71, Olympus, Tokyo, Japan).

### 4.5. Effects of ABT263 Concentration and Treatment Duration Period

ABT263 was dissolved in dimethyl sulfoxide (vehicle) for use in the experiments. To evaluate the effects of ABT263 concentration on the in vitro removal of senescent cells and damage to nonsenescent normal cells, both normal astrocytes and senescent astrocytes were treated with ABT263 at various concentrations for 24 h. To examine the effect of the ABT263 treatment duration on the in vitro removal of senescent cells, senescence-induced (OGD/R-treated) astrocytes were treated with 12 µM ABT263 for 0–72 h. To determine whether ABT263 treatment eliminates senescent astrocytes in vitro, cells were treated with either vehicle or 12 µM ABT263 for 96 h. The astrocyte culture medium was DMEM with high glucose supplemented with 10% (*v*/*v*) FBS and P/S.

### 4.6. Flow Cytometry Analysis for Apoptosis

To determine the percentages of apoptotic cells and viable cells, floating cells and adherent cells were collected from cultures. The cells were washed with cell staining buffer (Biolegend, San Diego, CA, USA) and resuspended in Annexin V binding buffer (Biolegend) and were stained with Annexin V (Biolegend) and PI (Biolegend) according to the manufacturer’s instructions. FACS Aria II (BD Biosciences, San Jose, CA, USA) and Flow Jo software (version 10, BD Biosciences) were used for evaluation.

### 4.7. Transient Middle Cerebral Artery Occlusion (MCAO) Modeling and Treatment

Male Sprague–Dawley (SD) rats (KOATECH, Seoul, Republic of Korea) aged 8–9 weeks and weighing between 250 and 300 g were used. All animal studies were performed in accordance with the guidelines and with the approval of the Institutional Animal Care and Use Committee of the Biomedical Research Institute of Seoul National University Hospital (IACUC No. 20-0165-S1A0 [1], date of approval: 18 August 2020). The focal ischemia-reperfusion model of the SD rats was established via the left intraluminal transient MCAO method. The MCAO model, which blocks blood flow to the middle cerebral artery, is a frequently used model in rodents to induce focal cerebral ischemia [55,56]. The induction of the transient intraluminal MCAO model was performed with the intra-arterial thread occlusion method as described previously [57,58,59]. Cerebral reperfusion was carried out by carefully removing the thread after 60 minutes of left MCAO, and the wound was sutured. The body temperature was maintained at 37 ± 0.5 °C by an electric heating blanket during the procedures, and monitoring was performed by a rectal probe. After transient MCAO induction, the experimental animals were randomly assigned to two groups: (1) the vehicle treatment group (Veh) and (2) the ABT263 (10 mg/kg) group (ABT). We administered ABT263 (Navitoclax, 10 mg/kg) or PBS as a vehicle intravenously three times. The first intravenous injection was performed immediately after transient MCAO model establishment, followed by administration at 24 h and then at 72 h after transient MCAO model development. Moreover, sham operations were performed in another group of SD rats as a negative control. The transient MCAO induction was performed in a total of 52 rats after power analysis and the minimal sample size calculation of the experiment.

### 4.8. Immunohistochemistry of Rat Brain Tissues

Brain tissues were collected and fixed in 4% paraformaldehyde solution. Sections of whole brain tissues with a thickness of 4 µm were dewaxed, rehydrated, and stained with antibodies against p16^INK4a^ (ab54210, 1:200, Abcam, Cambridge, UK), NOS2 (MAB9502, 1:20, R&D Systems, Minneapolis, MN, USA), MPO (ab208670, 1:1000, Abcam) and GFAP (ab7260, 1:1000, Abcam). After 24 h, the sections were washed with PBS and incubated with 2% rhodamine (TRITC)-conjugated AffiniPure goat anti-rabbit IgG (Jackson ImmunoResearch Labs, West Grove, PA, USA) and 2% fluorescein (FITC)-conjugated AffiniPure goat anti-mouse IgG (Jackson ImmunoResearch Labs) for 1 h. The nuclei were counterstained with 4′,6-diamidino-2-phenylindole (DAPI) after washing with PBS. The sections were mounted and examined using a fluorescence microscope (IX71, Olympus and SP8 X, Leica, Wetzlar, Germany). The percentage of positive area or cell number was analyzed using the Image J program (version 1.51k, National Institutes of Health, Bethesda, MD, USA). The minimal sample size calculation (power = 0.8, alpha = 0.05) estimated that six rats per group were required to be analyzed [60,61].

### 4.9. Measurement of Infarct Volume

Four days after transient MCAO, rats were anesthetized, and their brains were removed. The brain specimens were cut into 2-mm slices in the coronal direction and subjected to staining with 1% 2,3,5-triphenyltetrazolium chloride (TTC) under dark conditions at 37 °C prior, which covered the entire region of the transient MCAO (*n* = 8 per group). The cerebral infarct volume was measured using the images of the TTC stained sections by the Image J program (National Institutes of Health). Eight rats in each group were used after power analysis and the minimal sample size calculation (power = 0.8, alpha = 0.05) [60,61].

### 4.10. Neurological Function Assessment

Neurological function was evaluated to determine improvements in neurological deficits after treatment. The mNSS test and the limb-placing test were performed at 1, 3, 8, and 15 days following transient MCAO induction by an investigator who did not know the experimental grouping allocations. The mNSS is composed of an exercise test, tailing test, placement test, proprioceptive test, balance beam test, experiment of reflex loss and abnormal movement. The neurological deficits were assessed by a score scale of 0 to 18 (normal score, 0; maximal deficit score, 18) [59,62,63]. The two limb placing tests (LPT) evaluated the sensorimotor integration of the forelimb and hindlimb by checking the response to tactile and proprioceptive stimulations, as previously described; normal performance: 0 points, delayed or incomplete performance: 1 point, no performance: 2 points for both sides of the body (normal score, 0; maximal deficit score, 8) [59,62,64]. The group size was 6 rats in each experimental group according to power analysis and the minimal sample size calculation (power = 0.8, alpha = 0.05) [60,61].

### 4.11. Statistics

No animals or samples were excluded from the analysis. All statistical analyses were performed using Prism (version 8.02, GraphPad Software, San Diego, CA, USA). All data were expressed as means ± SD. Statistical significance was assessed using one-way ANOVA with Tukey’s posttest, two-way repeated measures ANOVA or the Mann–Whitney test.

## Data Availability

The datasets generated during and/or analyzed during the current study are available from the corresponding author on reasonable request.

## Supplementary Materials

The following are available online at https://www.mdpi.com/article/10.3390/ijms222111967/s1.
