# Supplementary material for: Senolytic Therapy for Cerebral Ischemia-Reperfusion Injury
Source: Int J Mol Sci. 2021 Nov 4;22(21):11967. doi: 10.3390/ijms222111967 (PMC8584561; doi:10.3390/ijms222111967)
Supplement: Supplementary file 1 [file ijms-22-11967-s001.zip › ijms-1443367-supplementary/211104 supplementary data_proofreading.pdf]

Supplementary File

Senolytic therapy for cerebral ischemia-reperfusion injury

Songhyun Lim, Tae Jung Kim, Young-Ju Kim, Cheesue Kim, Sang-Bae Ko*, Byung-Soo Kim*

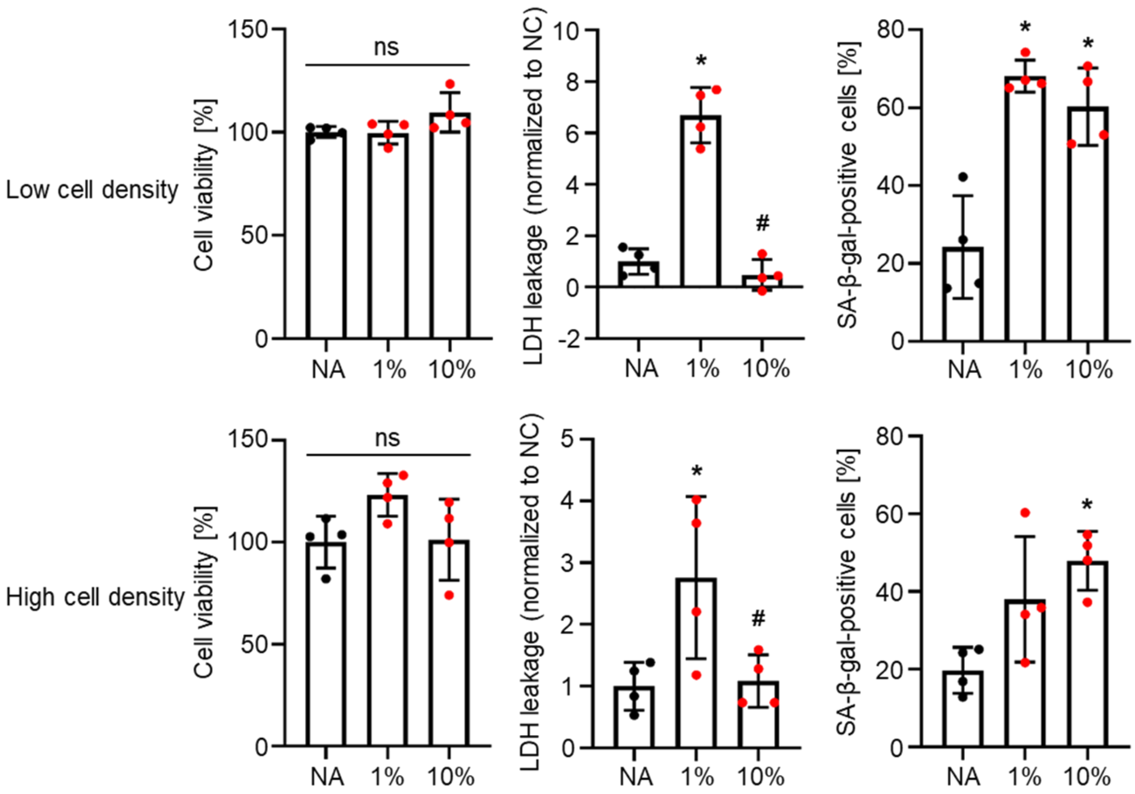

Figure S1. Effects of cell density and serum concentration of the culture medium on in vitro i nduction of senescence in OGD/R-injured astrocytes. Cell viability measured by MTT assay an d LDH release, and quantification of SA-β-gal staining of normal astrocytes (NA) and astrocyt es injured by OGD/R with two different serum concentrations (1% and 10%). n = 4 per group. *P<0.05 compared to NC, #P<0.05 compared to 1%. Data are presented as mean ± SD. One-w ay ANOVA with Tukey's post-test was used for statistical analysis.
